# New Chitosan/Iron Oxide Composites: Fabrication and Application for Removal of Sr^2+^ Radionuclide from Aqueous Solutions

**DOI:** 10.3390/biomimetics3040039

**Published:** 2018-12-04

**Authors:** Larisa Zemskova, Andrei Egorin, Eduard Tokar, Vladimir Ivanov, Svetlana Bratskaya

**Affiliations:** 1 Institute of Chemistry, Far Eastern Branch, Russian Academy of Sciences, prospect 100-letiya Vladivostoka, 159, Vladivostok 690022, Russia; zemskova@ich.dvo.ru (L.Z.); andrey.egorin@gmail.com (A.E.); d.edd@mail.ru (E.T.); 2 Far Eastern Federal University, Sukhanova str., 8, Vladivostok 690000, Russia; 3 Far East Geological Institute, Far Eastern Branch, Russian Academy of Sciences, prospect 100-letiya Vladivostoka, 159, Vladivostok 690022, Russia; d159327@yandex.ru

**Keywords:** composite materials, biosorbents, chitosan, iron oxides, strontium, adsorption

## Abstract

Here, we discuss the fabrication and problems of application of chitosan-based composite materials for the removal of hazardous metal ions from tap water and wastewater. The chitosan-based composites containing iron oxides for the uptake of Sr^2+^ ions were fabricated via a co-precipitation method with variation of the iron/chitosan ratio and pH of the medium. The morphology and composition of the fabricated sorbents were characterized using scanning electron microscopy–energy dispersive X-ray spectroscopy (SEM–EDX) and X-ray diffraction (XRD) analysis. We have shown that the suggested fabrication approach allows for a homogeneous distribution of the inorganic phase in the polymer matrix. Investigations of the sorption performance of the composites have shown that they are efficient sorbents for ^90^Sr radionuclides uptake from tap water. The composite sorbent containing amorphous iron oxide in a chitosan matrix and calcined at 105 °C showed the best sorption characteristics. We have also demonstrated that there is an optimal iron oxide content in the composite: with increasing oxide content, the efficiency of the sorbents decreases due to poor stability in solution, especially in alkaline media. The alternative approach yielding magnetic chitosan-based composites with sufficiently good sorption performance and stability in neutral and weakly alkaline media is suggested.

## 1. Introduction

The development of polymeric sorption materials, in which the natural polysaccharide chitosan is used as a matrix, is a rapidly developing field in the adsorption science. One of the attractive features of chitosan is that it can be used in adsorption processes in various physical forms, such as flakes, powders, nanoparticles, granules, membranes, and fibers/hollow fibers. To improve the mechanical properties and adsorption capacity, or even to prevent dissolution in acid medium of chitosan, numerous studies have been devoted to the chemical modification of chitosan by cross-linking or grafting with different polyfunctional agents [1,2,3]. Besides this, chitosan is used as a component of composite or hybrid materials with various inorganic substances or compounds [4]. In such composites, chitosan can be deposited on the surface of a porous material to provide high specific surface area and allow for better availability of active amino groups, improve mechanical properties, and thus optimize the efficiency of the target component uptake from the solution. In another approach, the inorganic component can be introduced into the chitosan solution, and a sorption material can then be obtained from the resulting mixture by known methods [5,6]. The most attractive method of composite fabrication is based on in situ formation of an inorganic component during chitosan precipitation [7]. In this case, a more homogeneous distribution of the inorganic ion exchanger in the polymer matrix is observed. In addition, chitosan can be used as a template for the fabrication of uniformly sized nanoparticles of metal oxides (for example, iron) for a broad range of applications, including sorption [8,9]. The use of oxide powders without a polymer matrix is associated with a number of problems, such as low mechanical strength and small particle size, which complicates subsequent separation of the sorbent from the solution, as well as high hydrodynamic resistance when used under dynamic conditions in sorption columns. However, the fabrication of mechanically strong composite materials offers a good solution to these problems.

Chitosan-based hybrid materials are becoming a promising alternative to conventional adsorbents and are used in water treatment and purification processes to remove such toxic pollutants as heavy metals, arsenic, radioactive contaminants, and organic impurities [3,5,6]. Natural clays, zeolites, perlite [5], and various metal oxides, which are traditional inorganic adsorbents for many pollutants, are used as inorganic components for composite fabrication. Miller and colleagues proposed a TiO_2_-impregnated chitosan as a sorbent for arsenic removal, which was obtained by adding anatase nanoparticles into chitosan solution followed by precipitation of sorbent granules in NaOH solution [10,11]. The composite sorbent Al_2_O_3_/chitosan for arsenic uptake was fabricated by mixing a gel of chitosan and aluminum oxide with subsequent treatment of the paste with alkali [12]. Composite sorbents for As(III) and As(V) obtained by depositing iron oxides on chitosan flakes or in the form of chitosan granules containing oxide were reported in [13]. 

Iron oxides are universal sorbents for many pollutants, including radionuclides. The most attractive type of such sorbents comprises magnetic sorbents, including magnetic chitosan-containing composites [6,7]. The use of such sorbents for metal ion uptake allows combining the purification process with that of the sorbent magnetic separation.

The uptake of uranium using chitosan and chitosan-based materials is known to be considered as one of the most important applications of chitosan [14]. This also includes the fabrication of a variety of magnetic chitosan resins for uranium sorption [15,16,17,18,19]. In magnetic sorbents, chitosan plays a special role. Magnetic nanoparticles are highly chemically active and easily oxidized in air, which leads to a loss of magnetic properties and a decrease in the degree of dispersity. When magnetic particles are coated with chitosan, not only does their susceptibility to oxidation decrease (increasing the shelf-life of the sorbents), but also their tendency to aggregation [7]. Interestingly, magnetic chitosan-based sorbents can be further modified via the introduction of specific functional groups to enhance the selectivity or via polymer cross-linking to improve the mechanical properties or prevent solubility in acidic media [15,16,17,18,19]. 

In order to remove hazardous cesium and strontium radionuclides, in addition to uranium, composite chitosan-based sorbents can be obtained. Chitosan poorly adsorbs alkali and alkali earth metals [14]; therefore, chitosan loaded with transition metal ferrocyanides selective to cesium is used to remove cesium radionuclides [20,21]. Sorbents containing iron oxide [22], including those embedded in the chitosan matrix [23], are considered as sorbents for strontium.

The present work was aimed at the development of methods of fabrication of composite sorbents based on iron oxides homogeneously distributed in a chitosan matrix and the investigation of their sorption performance for the removal of strontium ions from highly diluted solutions.

## 2. Materials and Methods

Chitosan was purchased from JSC “Vostok-Bor” (Dalnegorsk, Russia); the degree of acetylation was 0.25, and the viscosity-averaged molecular weight was 250 kDa. Iron(III) chloride (FeCl_3_ × 6H_2_O), iron(II) sulphate (FeSO_4_ × 7H_2_O), ammonium hydroxide (NH_4_OH), hydrochloric acid (HCl), strontium (stable) chloride (SrCl_2_ × 6H_2_O), and sodium hydroxide (NaOH) were purchased from Nevareaktiv (Saint Petersburg, Russia). All chemicals were of analytical grade and were used as received without further purification.

Nonmagnetic sorbents were obtained via addition of the solutions of Fe(III) salt into a 1% solution of chitosan (in 0.1 M HCl) at the Fe/chitosan ratios 1:1, 2:1, and 4:1 (g/g)—amorphous sorbents 1A, 2A, and 3A, respectively. The NH_4_OH solution was added to the obtained mixture until neutral reaction. Magnetic sorbents were obtained via addition of the mixture of solutions of Fe(III) and Fe(II) salts (molar ratio 1:2) into a 1% solution of chitosan (in 0.1 M HCl) at the Fe/chitosan ratio 1:1 (g/g). The NH_4_OH solution was added to the obtained mixture until weakly alkaline reaction, pH 8–9 (sorbent 2M), or neutral reaction, pH 7.0 ± 0.5 (sorbent 3M). A magnetic powder of iron oxide (Fe_3_O_4_) (sorbent 1M) without chitosan was obtained via addition of NH_4_OH solution to the mixture of the solutions of Fe(II) and Fe(III) salts (ratio 2:1 mol/mol) until neutral reaction. Precipitates were washed with distilled water, dried, and calcined at 105 °C for 1 h.

The morphology of the composite materials and distribution of the inorganic component in the bulk were investigated using a Lyra3 XMH (Tescan, Brno, Czech Republic) scanning electron microscope equipped with an AZtecEnergy energy dispersive X-ray (EDX) microanalyzer automated with an X-Max80 detector (Oxford Instruments, Abingdon, UK). X-ray diffraction (XRD) analysis was carried out using a SmartLab diffractometer (Rigaku, Tokyo, Japan) with Cu Kα radiation in the 2θ range from 2° to 80°. 

The efficacy of ^90^Sr uptake was studied in tap water at a sorbent/liquid ratio of 1:1000 g/mL. Air-dried sorbent with a bead size of 0.1–0.2 mm was added to tap water (pH 6.5) spiked with ^90^Sr radionuclide with an initial activity of 800 Bq/mL. After 7 days, the solution was separated from the sorbent and filtered through blue ribbon filter paper with a pore size of 3 µm, and the equilibrium activity was measured using a Tri-Carb 2910TR liquid scintillation alfa, beta-spectrometer (Perkin Elmer, Waltham, MA, USA). Isotherms of Sr^2+^ sorption on virgin Fe_3_O_4_ and magnetic sorbent 3M without spiking with ^90^Sr at a sorbent/liquid ratio of 1:1000 (g/mL) were determined. The equilibrium strontium concentration was determined after 7 days by atomic absorption spectrometry (AAS) using a Solaar M6 spectrometer (Thermo, Waltham, MA, USA). The sorption isotherms were fitted with Freundlich (Equation (1)) and Langmuir (Equation (2)) models using SciDAVis software (version 1.23; [24]):

(1) $$G=K_{f}\times C^{n}$$

(2) $$G=G_{max}\times \frac{K_{l}\times C}{1+K_{l}\times C}$$

where *G_max_* is the maximum sorption capacity (mg/g), *C* is the equilibrium concentration of Sr (mg/L), *K_f_* is the Freundlich constant, *K_l_* is the Langmuir constant, and *n* is the coefficient related to the heterogeneity of the sorption centers. 

The efficiency of the ^90^Sr uptake with different sorption materials was estimated via distribution coefficients calculated using Equation (3):

(3) $$K_{d}=\frac{A_{0}-A_{1}}{A_{1}}\times \frac{V}{m}$$

where *K_d_* is the distribution coefficient of ^90^Sr (mL/g); *A*_0_ and *A*_1_ are the initial and equilibrium activities of the spiked tap water (Bq/mL), respectively; *V* is the volume of spiked tap water; and *m* is the weight of the sorbent (g).

^90^Sr kinetic curves were obtained using tap water spiked with a radionuclide at a V/m ratio of 1000 mL/g. The sorption value was calculated according to Equation (4):

(4) $$S=\left(1-\left(\frac{A_{1}}{A_{0}}\right)\right)\times 100$$

where *A*_0_ and *A*_1_ are the initial and equilibrium activities of the spiked tap water (Bq/mL), respectively.

The zero charge point (pH_PZC_) of the sorbent was determined by a static method as follows: a quantity of 0.2 g of the sorbent was immersed into 0.1 M NaCl solutions with different pH values. The initial pH value was maintained by the addition of 0.05 M NaOH and HCl solutions. Sorbents were equilibrated under constant stirring for 36 h, then separated by filtration, and the equilibrium pH value was measured. The pH_PZC_ was assigned to the pH value corresponding to the horizontal inflection on the curve showing dependence of equilibrium pH on initial pH values [25]. The mechanical strength of sorbents was studied under dynamic conditions when passing 5 L of tap water at a flow rate of 75 mL/h through a column diameter of 10 mm, with sorbent volume of 1 mL and granulation of 0.1–0.2 mm. After water passing, the material was dried until constant weight at 105 °C and sieved again, and the weight of sorbent particles of sizes less than 0.1 mm was estimated.

The specific surface area was measured by low-temperature N_2_ sorption at 77 K using an Autosorb iQ automated sorption analyzer (Quantachrome Instruments, Boynton Beach, FL, USA). Prior to the experiment, samples were degassed at 105 °C for 6 h. The specific surface area was calculated via the Brunauer–Emmett–Teller (BET) method. 

## 3. Results

### 3.1. Sorbent Characterization 

**Figure 1:**
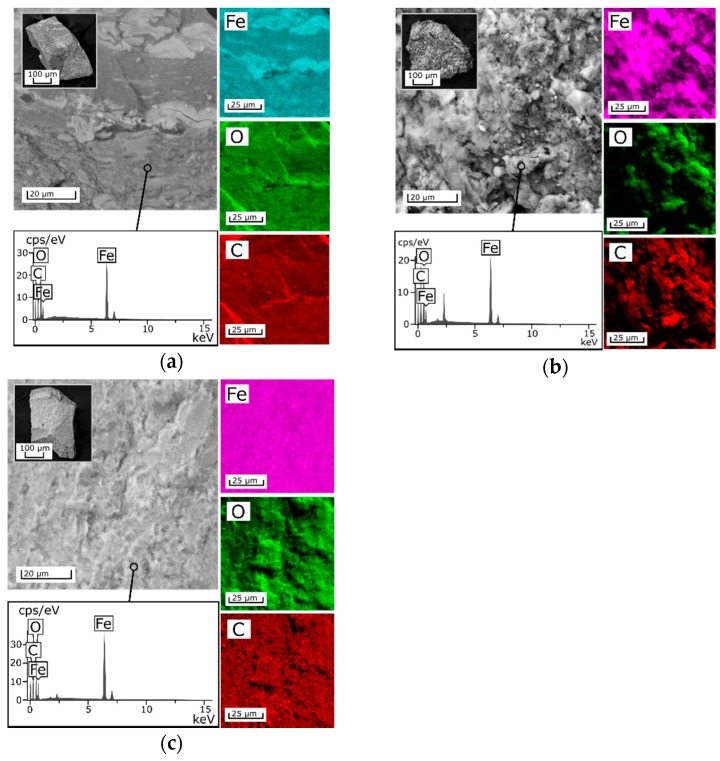
Scanning electron microscopy–energy dispersive X-ray spectroscopy (SEM–EDX) analysis of composite sorbents. Sorbents (**a**) 1A; (**b**) 2M; and (**c**) 3M.

Scanning electron microscopy (SEM) images with the results of the EDX analysis of chitosan-based sorbents containing iron oxides are shown in Figure 1. The SEM images for sorbents 2A and 3A did not have significant differences from those of 1A, 2M, and 3M and were therefore not shown in Figure 1. The sorbent beads obtained by crushing and subsequent sieving of fractions with a given particle size have an irregular shape. The EDX mapping confirms that all elements are homogeneously distributed in both the magnetic and nonmagnetic composite sorbents.

**Figure 2:**
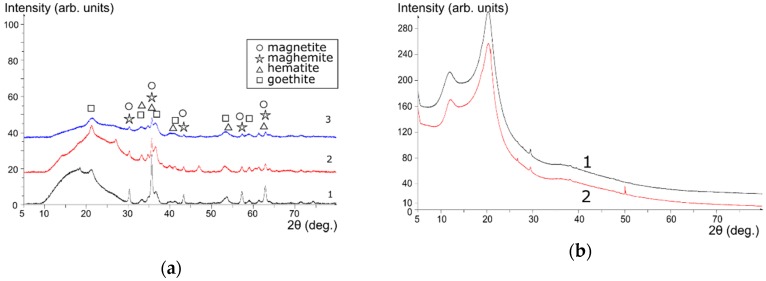
X-ray diffraction (XRD) patterns of composite materials and iron oxide. Sorbents (**a**) 3M (1); 2M (2); and virgin magnetic powder Fe_3_O_4_, 1M (3). (**b**) Unmodified chitosan (1); chitosan calcined at 110 °C (2).

The results of the XRD analysis of magnetic sorbent powders are shown in Figure 2a. Using the PDF-2 database (2017 release; [26]), the following phases were identified: Fe_3_O_4_ maghemite (card number 00-039-1346), Fe_3_O_4_ hematite (card number 01-089-0596), FeO(OH) goethite (card number 00-029-0713), and Fe_3_O_4_ magnetite (card number 01-089-0691). Figure 2b shows unmodified chitosan and chitosan calcined at 110 °C for comparison. 

### 3.2. Evaluation of Sorption Properties 

**Figure 3:**
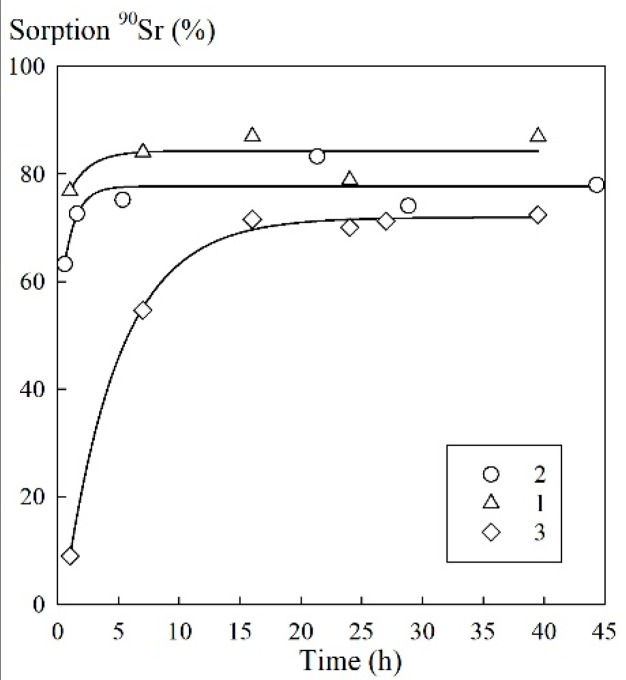
Kinetic curves of ^90^Sr sorption from tap water. Sorbents 1A (1); 3M (2); and 1M (3).

The results of kinetic properties studies are shown in Figure 3 for a sorbent/tap water ratio of 1:1000 (g/mL).

The results of the comparative investigation of the sorption of the ^90^Sr radionuclide from tap water on virgin iron oxide and on magnetic and nonmagnetic amorphous chitosan-based sorbents are presented in Table 1. Table 1 shows the relationship between the sorption efficiency expressed as K_d_^90^Sr and composition and the preparation conditions of the chitosan-based composite. Noteworthy, only iron oxide works as an active component of the composite. Thus, to estimate the difference in its performance in the form of free powder and as a component of the composite, ^90^Sr distribution coefficients were also calculated taking into account the content of the inorganic phase in the composites.

**Figure 4:**
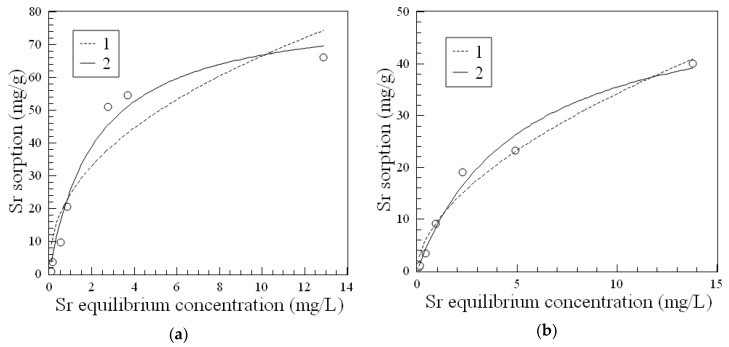
Isotherms of Sr^2+^ sorption for sorbents (**a**) Fe_3_O_4_ and (**b**) 3M. Circles represent the experimental data; fit lines are computed using the Freundlich and Langmuir equations (Equations (1) and (2), respectively).

Figure 4 shows the isotherm of Sr^2+^ sorption from distilled water on virgin Fe_3_O_4_ and 3M sorbent and the results of experimental data fitting using the Freundlich and Langmuir equations (Equations (1) and (2), respectively). The obtained sorption isotherm according to the Giles classification can be attributed to the L-type or H-type, which indicates high affinity of the 3M material with respect to Sr^2+^ ions in the region of low concentration.

Table 2 summarizes the parameters of the Freundlich and Langmuir equations for the isotherms of Sr^2+^ sorption on Fe_3_O_4_ and the 3M composite, which show that the Langmuir model better describes the experimental data for both materials. 

### 3.3. Evaluation of Sorbent Stability in Solution 

The evaluation of composite sorbent stability in solution was carried out in the process of sorption properties investigation (i.e., during the 7 days of ^90^Sr radionuclide uptake from tap water).

**Figure 5:**
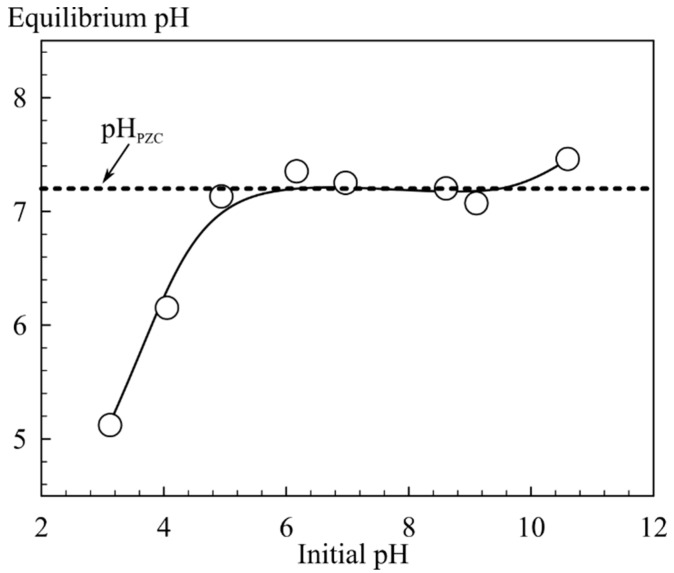
Point of zero charge (pH_PZC_) of the sorbent 3M.

**Figure 6:**
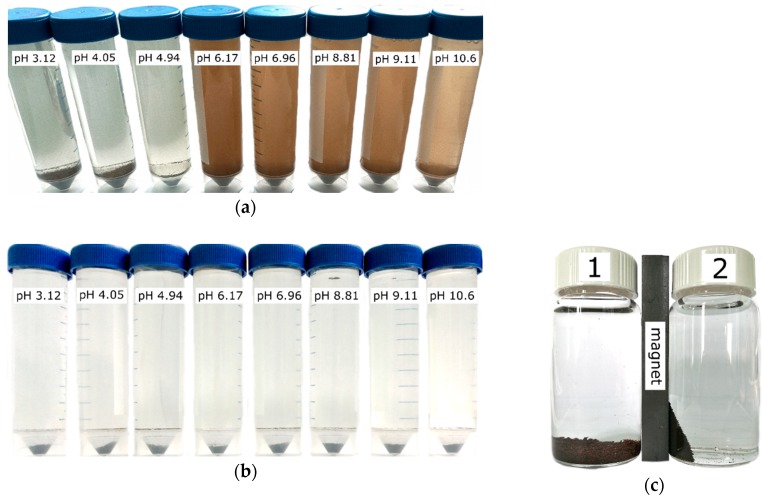
Visual evaluation of the composite sorbent suspensions in 0.1 M NaCl solution with different pH values after 1 week (sorbent weight is 0.2 g, volume of solution is 50 mL). Sorbents (**a**) 1A; (**b**) 3M; and (**c**) their magnetic properties (1A (1); 3M (2)).

In addition, the stability of the sorbents was evaluated when determining the point of zero charge of the surface in 0.1 M NaCl solution. The experimental results from the determination of the pH_PZC_ are shown in Figure 5. They are presented as a dependence of the pH of the filtered solutions after equilibration of the sorbent in solution for 36 h on the pH values of the initial solutions. A visual assessment of the stability of the sorbents in NaCl solutions is shown in Figure 6. 

## 4. Discussion

Investigations of the composite sorbents’ morphology revealed that beads of the 3M sorbent calcined at 105 °C have denser monolithic structure compared to the 2M sorbent (Figure 1b,c). The layers observed in the SEM image of the 2M sorbent determine a more porous structure of this composite. To confirm this fact, the specific surface area of the composites was measured and was found to be 39.7, 58.9, and 14.8 m^2^/g for samples 1A, 2M, and 3M, respectively. In terms of texture and morphology, the amorphous 1A composite is more similar to the 3M sorbent (Figure 1a). According to the SEM–EDX mapping (Figure 1), the proposed method of composite sorbent fabrication, in which precipitation of iron oxides occurs simultaneously with the precipitation of chitosan, yields hybrid materials with an active inorganic phase homogeneously distributed in the polymer matrix. 

According to the XRD data, precipitation of iron oxides with ammonia solutions from the mixture of Fe(III) and Fe(II) salts yields precipitates which after calcination at 105 °C have nearly the same composition, regardless of the chitosan presence (Figure 2a). Goethite, hematite, maghemite, and possibly magnetite were found in the composition of sorbents. During the precipitation of the Fe(III) salt with a solution of NH_4_OH, amorphous Fe(III) oxide/hydroxide was formed. As a result, the 3M composite presented the best magnetization and was therefore easier to separate from the solution by magnetic separation compared to the 2M composite. Noteworthy, heating of chitosan up to 110 °C did not lead to changes in its crystalline structure. Instead, when precipitated together with iron oxides, amorphization of the polymer occured in the composite.

Investigations of the ^90^Sr uptake from tap water by magnetic and nonmagnetic amorphous composites in comparison with Fe_3_O_4_ powder showed that virgin Fe_3_O_4_ and amorphous composite 1A are the most effective sorbents (Table 1). The high sorption efficiency of these materials can be explained by the sorbent gradual dispergation during the adsorption tests with stirring, which increased the sorbent specific surface area and simplified the access to the sorption centers. In the case of Fe_3_O_4_, the sorbent degradation showed a mechanical character and was accompanied by the formation of smaller particles which, however, could be separated from the solution on a cellulose filter with a pore size of 3 μm. The destruction of the composite sorbent 1A presented a different nature and was associated with the hydrolysis and formation of small flakes of brown or orange color and poorly filterable colloidal particles, probably of Fe(OH)_3_, leading to visually detectable opalescence of the solution. The destruction of the sorbent 1A occured at pH 6.17 and above (Figure 5) which limited the application of this material despite the highest value of the distribution coefficient (Table 1). The surface of iron oxide showed a pH_PZC_ in the region close to neutral pH, suggesting that hydrolysis in alkaline solutions contributes to a negative surface charge. Therefore, more efficient sorption of Sr^2+^ ions is observed in alkaline solutions [22]. An increase of inorganic phase content in the nonmagnetic composite sorbents (2A, 3A) (Table 1) lead to a noticeable intensification of the destruction process due to hydrolysis and release of ^90^Sr in the pseudocolloid state (sorbed on unfilterable colloidal particles of the partially degraded composite). This resulted in a poor sorption performance of composites with the increase of the inorganic phase content in a series from 1A to 3A (Table 1). In the process of testing the strength of the sorbents, it was found that 6.6% of the magnetic powder (1M) is destroyed under experimental conditions compared to the destruction of 2.4% of composite sorbent 3M and 0.5% of 2M.

Based on the kinetics of the process of sorption during the removal of microconcentrations of ^90^Sr, the time of attainment of the sorption equilibrium under static conditions was 5 h for the sorbents 1A and 3M. For the sorbent 1M, this time was 15 h (Figure 5).

Although the distribution coefficient for ^90^Sr on the magnetic 3M composite is somewhat lower compared to the best of nonmagnetic amorphous composite 1A (Table 1), it showed significantly high sorption capacity toward Sr^2+^ ions (Figure 3). The parameters of the Langmuir model, which provides the most accurate fit to the experimental data of Sr^2+^ sorption isotherms, showed that the 3M sorbent has high affinity toward Sr^2+^ ions as well (Table 2). A comparison of the Langmuir constants and the maximum sorption capacities of the virgin Fe_3_O_4_ and the 3M composite (Table 2), taking into account the content of the inorganic phase in the 3M sorbent, allows for the conclusion that the fabrication method of the 3M composite preserves the sorption characteristics of iron oxide but provides a material with better stability in solution.

## 5. Conclusions

The use of natural polymer chitosan as a matrix for the synthesis of iron oxide sorbents appears to be a useful, cost-effective methodology. Iron oxides are very effective sorbents for a number of elements, including strontium [22]. However, the poor mechanical properties and stability of iron oxides in solution, especially due to peptization in alkaline media, are unsatisfactory for their direct application for water treatment [23]. Here, we have demonstrated that chitosan-based composites, which were fabricated via co-precipitation of iron oxides with chitosan, have the necessary technological and functional characteristics to make them applicable in water treatment technologies. 

## Figures and Tables

**Table 1:** Distribution coefficients of ^90^Sr for virgin iron oxide and composite materials.

Sorbent	Fe_3_O_4_	1A	2A	3A	2M	3M
*K_d_*^90^Sr × 10^−3^ (mL/g)	36.5	9.5	4.9	2.0	2.4	2.4
Inorganic phase content (wt %)	100	42	53	61	42	42
*K_d_*^90^Sr × 10^−3^ calculated per inorganic phase (mL/g)	36.5	22.7	9.2	3.3	5.7	5.7

*K_d_*: Distribution coefficient of ^90^Sr.

**Table 2:** Parameters of Freundlich and Langmuir equations for the isotherms of Sr^2+^ sorption on virgin Fe_3_O_4_ and the magnetic composite 3M.

Sorbent	Freundlich Equation			Langmuir Equation		
	*K_f_*	*n*	*R* ^2^	*G*_max_	*K_l_*	*R* ^2^
3M	9.5 ± 1.2	1.8 ± 0.2	0.97	54 ± 5	0.2 ± 0.04	0.98
Fe_3_O_4_	24 ± 5	2.3 ± 0.5	0.86	81 ± 7	0.5 ± 0.1	0.97

*G*_max_: Maximum sorption capacity mg/g (Sr^2+^/sorbent); *K_L_*: Langmuir constant; *K_f_*: Freundlich constant; *n*: Heterogeneity coefficient of sorption centers; *R*^2^: Correlation coefficient.

## References

[1] Guibal E. (2004). Interactions of metal ions with chitosan-based sorbents: A review. Sep. Purif. Technol..

[2] Adarsh J.K., Madhu G. (2014). A comparative study on metal adsorption properties of different forms of chitosan. Int. J. Innov. Res. Sci. Eng. Technol..

[3] Elwakeel K.Z. (2010). Environmental application of chitosan resins for the treatment of water and wastewater: A Review. J. Dispers. Sci. Technol..

[4] Crini G. (2005). Recent developments in polysaccharide-based materials used as adsorbents in wastewater treatment. Prog. Polym. Sci..

[5] Wan Ngah W.S., Teong L.C., Hanafiah M.A.K.M. (2011). Adsorption of dyes and heavy metal ions by chitosan composites: A review. Carbohydr. Polym..

[6] Wang J., Chen C. (2014). Chitosan-based biosorbents: Modification and application for biosorption of heavy metals and radionuclides. Bioresour. Technol..

[7] Reddy D.H.K., Lee S.-M. (2013). Application of magnetic chitosan composites for the removal of toxic metal and dyes from aqueous solutions. Adv. Colloid Interface Sci..

[8] Nidhin M., Indumathy R., Sreeram K.J., Nair B.U. (2008). Synthesis of iron oxide nanoparticles of narrow size distribution on polysaccharide templates. Bull. Mater. Sci..

[9] Janardhanan S.K., Ramasamy I., Nair B.U. (2008). Synthesis of iron oxide nanoparticles using chitosan and starch templates. Transit. Met. Chem..

[10] Miller S.M., Spaulding M.L., Zimmerman J.B. (2011). Optimization of capacity and kinetics for a novel bio-based arsenic sorbent, TiO_2_-impregnated chitosan bead. Water Res..

[11] Miller S.M., Zimmerman J.B. (2010). Novel, bio-based, photoactive arsenic sorbent: TiO_2_-impregnated chitosan bead. Water Res..

[12] Boddu V.M., Abburi K., Talbott J.L., Smith E.D., Haasch R. (2008). Removal of arsenic (III) and arsenic (V) from aqueous medium using chitosan-coated biosorbent. Water Res..

[13] Gupta A., Chauhan V.S., Sankararamakrishnan N. (2009). Preparation and evaluation of iron–chitosan composites for removal of As(III) and As(V) from arsenic contaminated real life groundwater. Water Res..

[14] Muzzarelli R.A.A. (2011). Potential of chitin/chitosan-bearing materials for uranium recovery: An interdisciplinary review. Carbohydr. Polym..

[15] Wang J., Peng R., Yang J., Liu Y., Hu X. (2011). Preparation of ethylenediamine-modified magnetic chitosan complex for adsorption of uranyl ions. Carbohydr. Polym..

[16] Xu J., Chen M., Zhang C., Yi Z. (2013). Adsorption of uranium (VI) from aqueous solution by diethylenetriamine-functionalized magnetic chitosan. J. Radioanal. Nucl. Chem..

[17] Zhou L., Jia Y., Peng J., Liu Z., Al-Zaini E. (2014). Competitive adsorption of uranium(VI) and thorium(IV) ions from aqueous solution using triphosphate-crosslinked magnetic chitosan resins. J. Radioanal. Nucl. Chem..

[18] Mahfouz M.G., Galhoum A.A., Gomaa N.A., Abdel-Rehem S.S., Atia A.A., Vincent T., Guibal E. (2015). Uranium extraction using magnetic nano-based particles of diethylenetriamine-functionalized chitosan: Equilibrium and kinetic studies. Chem. Eng. J..

[19] Galhoum A.A., Mahfouz M.G., Gomaa N.M., Vincent T., Guibal E. (2017). Chemical modifications of chitosan nano-based magnetic particles for enhanced uranyl sorption. Hydrometallurgy.

[20] Vincent C., Hertz A., Vincent T., Barré Y., Guibal E. (2014). Immobilization of inorganic ion-exchanger into biopolymer foams—Application to cesium sorption. Chem. Eng. J..

[21] Vincent T., Vincent C., Barré Y., Guari Y., Saout G.L., Guibal E. (2014). Immobilization of metal hexacyanoferrates in chitin beads for cesium sorption: Synthesis and characterization. J. Mater. Chem. A.

[22] Liu C.-H., Shih Y.-J., Huang Y.-H., Huang C.-P. (2014). Kinetic and thermodynamic studies for adsorptive removal of Sr^2+^ using waste iron oxide. J. Taiwan Inst. Chem. Eng..

[23] Chen Y., Wang J. (2012). Removal of radionuclide Sr^2+^ ions from aqueous solution using synthesized magnetic chitosan beads. Nucl. Eng. Des..

[24] SciDAVis Download. https://sourceforge.net/projects/scidavis/.

[25] Milonjić S.K., Ruvarac A.L., Šušić M.V. (1975). The heat of immersion of natural magnetite in aqueous solutions. Thermochim. Acta.

[26] International Centre for Diffraction Data. http://www.icdd.com/index.php/pdf-2/.

